# Evaluation of accuracy of three-dimensional printing and three-dimensional miniplates in treatment of anterior mandibular fractures: a prospective clinical study

**DOI:** 10.1186/s12903-025-05935-1

**Published:** 2025-04-28

**Authors:** Mazen Tharwat Abou Elkhier, Mohamed Elsayed Saber, Ahmed Ossama Sweedan, Adham Elashwah

**Affiliations:** Oral and Maxillofacial Surgery, Faculty of Dentistry, Alexandria University, Alexandria, Egypt

**Keywords:** Virtual planning. 3D printing, 3D miniplates, Mandibular fracture

## Abstract

**Background:**

There are strong torsional forces acting on the anterior mandible fractures. Maxillofacial surgery makes extensive use of digital technology, and three-dimensional printing is now an integral element of the workflow in several areas of oral and maxillofacial surgery. Three-dimensional (3D) miniplates had been proposed by several researchers as a good option for mandibular fracture fixation. This clinical trial was conducted to evaluate the accuracy of virtual planning in anterior mandibular fixation by comparing postoperative outcomes with preoperative virtual planning and to evaluate bone healing by measuring bone density after using pre-bent 3D miniplates on a 3D model to complete a 3D workflow and to fix the mandible in 3D.

**Methods:**

15 Patients with anterior mandibular fractures were included in the study. All patients underwent computer tomography (CT) scan, and the data were imported into Mimics software. The unaffected healthy side was mirrored to the fractured side. Bone fixation three-dimensional plates were prebent and adapted on the model printed by the three-dimensional printing machine, submitted to sterilization, and were used for bone reduction and fixation. An immediate postoperative CT scan was taken to evaluate the accuracy of virtual planning and after 3 months for evaluation of bone healing.

**Results:**

Clinical observation revealed good stable occlusion, and there was no significant difference between the postoperative three-dimensional image of the mandible and the virtually reduced mandible in the preoperative plan, as there was no significant difference between the length and width between anatomical landmarks of the virtually reduced mandible and the postoperative plan (*p* > 0.05). The bone density measured by Hounsfield units (HU) measured on CT images after 3 months revealed good bone healing as compared to immediate postoperative values (P value < 0.05).

**Conclusion:**

Digital workflow provides an accurate method for the reduction and fixation of anterior mandibular fractures. Also, 3D miniplates provide a good option for symphyseal and parasymphyseal fractures despite their limitations, as in some cases, like comminuted fractures and fractures in and around the mental foramen.

**Trial registration:**

This clinical trial was registered at Alexandria university, on 02/11/2021 under the registration number 0308 − 10/2021. All procedures involving human participants were performed in accordance with Research Ethics Committee, Faculty of Dentistry, Alexandria University under IRB No 00010556 and IORG No 0008839. The current study was retrospectively registered at ClinicalTrials.gov with the identification number NCT06898736 on 27/3/2025. However, all study protocols were predefined, with no deviations from the original methodology.

## Background

The mandible is the largest and most prominent bone in the maxillofacial region. Facial fractures frequently involve the mandible [1]. Rigid internal fixation combined with maxillomandibular fixation is the standard surgical procedure for treating mandibular fractures [2]. Maxillofacial surgery currently makes extensive use of digital technology in.

areas like surgical navigation, titanium plate preforming technology, and 3D digital guide plate technology [3–5].

Monocortical miniplates that are easily adaptable are used in Champy’s semi-rigid fixation technique, along with an “ideal osteosynthesis line” to counteract the developing masticatory forces [6]. The location of the bone plate fixation must provide the fixation method that is most stable in relation to the line of tension at the base of the alveolar process. One plate is often used for fractures that are posterior to the foramen, and two plates are typically used for fractures anterior to the foramen [6, 7].

Due to muscles working against each other, the anterior mandible fractures are subject to strong torsional forces. Some researchers have suggested using the three-dimensional (3D) miniplates as a viable option for fixation [8].

To ensure successful osteosynthesis with a minimally invasive approach, three-dimensional (3D) miniplate systems can be used. The system comprises two miniplates joined by interconnecting crossbars and fixed to the bone with monocortical screws. When placed in the fracture line, the 3D miniplate system ensures fracture stabilization, regardless of plate thickness and geometry. The placement of screws in a square pattern on either side of the fracture creates a solid platform that increases resistance to twisting and rotation of the longitudinal axis of the plate [9–11].

The stability of occlusion after using 3D miniplates may be due to the box-like configuration, providing rigid fixation of fractures that prevent Buccolingual splaying and gap formation at the fracture site and subsequent occlusal discrepancy; this is the advantage of 3D miniplates over 2D miniplates [6].

The surgeon is now able to use 3D segmentation and mirroring methods, which are highly effective in simulating the pre-traumatized anatomy at a minimal cost, using advanced open-source software programs [12].

The idea behind 3D printing in the medical industry is to take anatomical scans utilizing imaging methods like computed tomography (CT) and magnetic resonance imaging (MRI). The images from these methods will be saved in a common format, such as the Digital Imaging and Communications in Medicine (DICOM) format, and then with the aid of computer-aided design (CAD) software, a virtual 3D prototype with Standard Tessellation Language (STL) format will be created to enable 3D printing and the deposition of the material layer by layer to achieve the final structure [13–15].

Thus, in the present study, the goal was to assess the accuracy of virtual planning and pre-bent 3D miniplates in anterior mandibular fixation by comparing postoperative outcomes with preoperative virtual reduction and to evaluate the clinical outcomes and bone density after fixation with 3D miniplates.

## Materials and methods

**Fig. 1:**
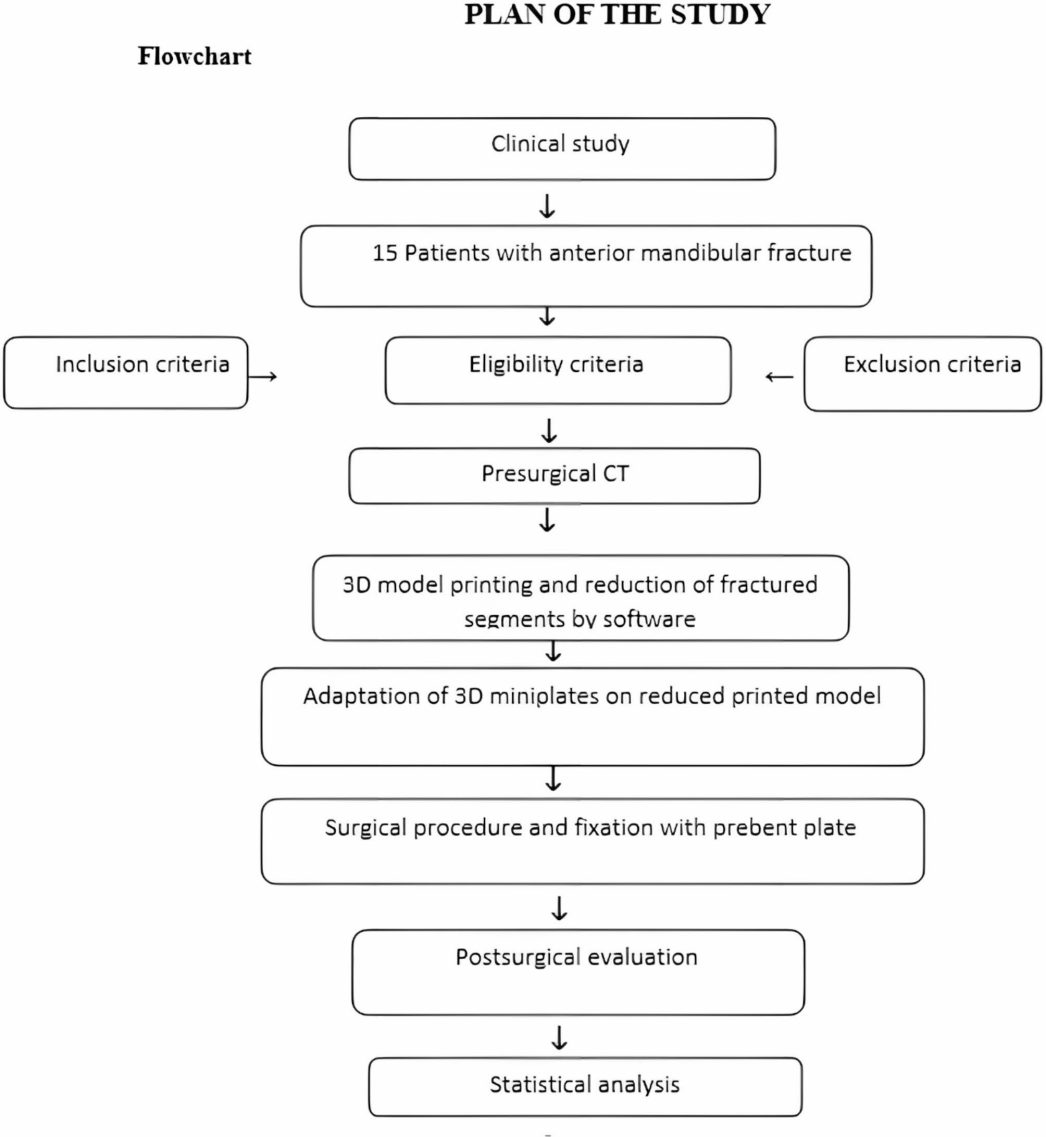
A flowchart summarizing the study design

A flowchart summarizing the study workflow and study design is shown in Fig. 1.

### Sample size Estimation

Patients with anterior mandibular fractures were selected from the outpatient clinic of the oral and maxillofacial surgery department, and the surgical procedures were performed under general anesthesia. Sample size was estimated using G-Power version 3.1.9.2 (Faul, Erdfelder, Lang. & Buchner. 2007) [16]. A minimal total hypothesized sample size of 15 patients with anterior mandibular fractures was needed to assess the mean difference in the hardware fixation time and to test the efficacy of virtual planning and 3D printing and 3D miniplates for fixation of anterior mandibular fractures, taking into consideration a 95% confidence level and 80% power using a paired t-test.

### Ethical considerations

This study was performed after gaining ethical approval from the Research Ethics Committee, Faculty of Dentistry, Alexandria University with the number 0308 − 10/2021. Before starting the study, objectives, risks, and benefits were explained to the patients, and they received both oral and written information about the benefits and risks of the interventions, and signed, informed consent was obtained prior to treatment.

The patients were followed up in the outpatient clinic of the Oral and Maxillofacial Surgery Department, Faculty of Dentistry. All personal information of the patients was recorded, and direct contact with the patients and their relatives was established using telephone numbers to ensure ease of recall and to provide keen and up-to-date follow-up.

The participating patients in this study were chosen according to the following criteria:

### Inclusion criteria

1. Recent anterior mandibular fractures (within one week).
2. Adult patients of both genders (18–50 years old).
3. Systematic healthy patients.
4. Radiographic evidence of anterior mandibular fracture.

### Exclusion criteria

1. Medically compromised patients contraindicated for general anesthesia and bone surgery.
2. Comminuted fractures.
3. Bilateral anterior mandibular fractures.
4. Previously treated fractures.
5. Pathological fractures and bone diseases.

### Plate specifications

**Fig. 2:**
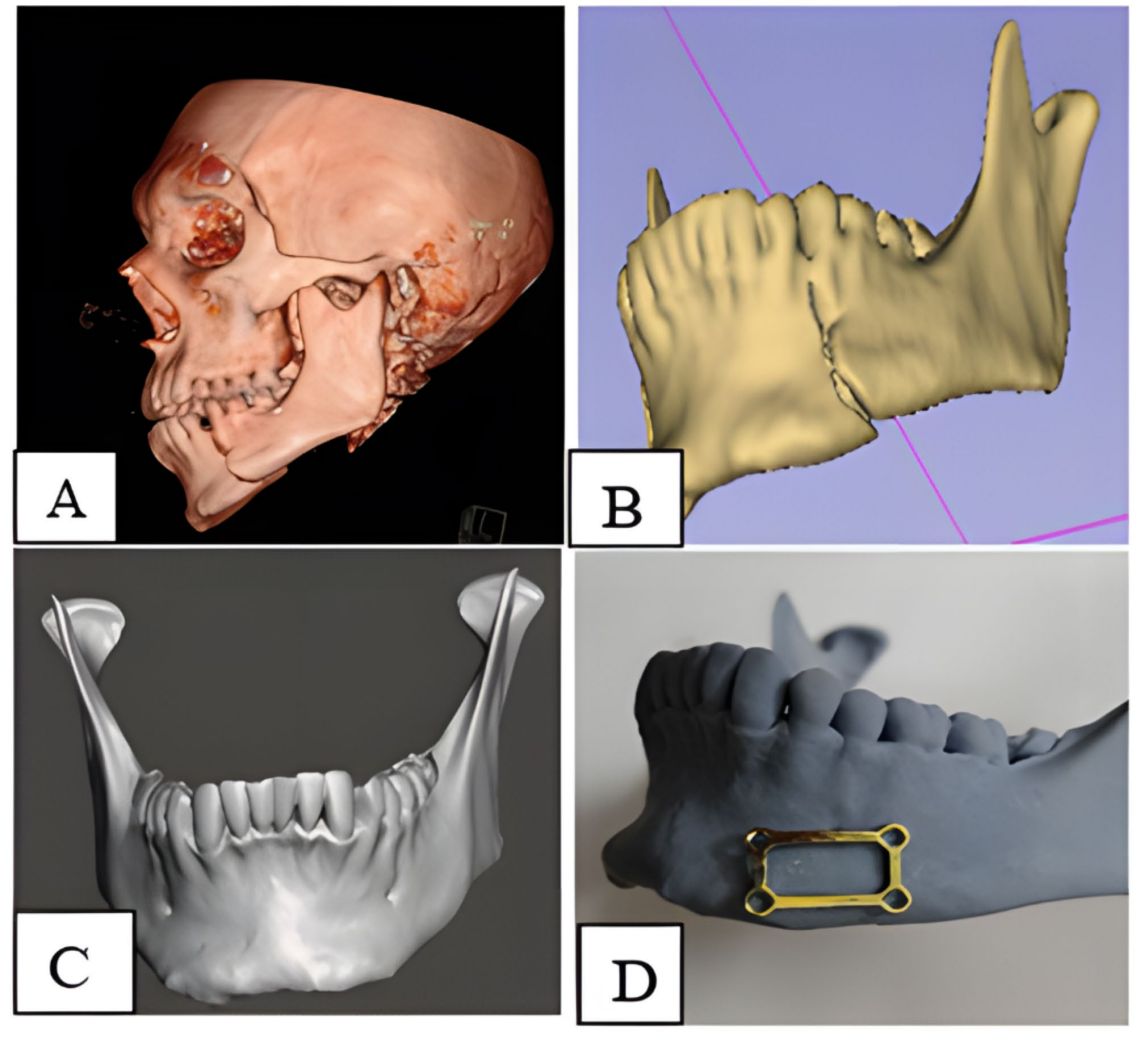
Preoperative surgical planning. **(A)** 3D reconstructed image from CT. **(B)** segmented mandible from CT. **(C)** virtually reduced mandibular fracture. **(D)** printed 3D model with prebent miniplate

The plates were designed and manufactured from titanium material grade 4. Three-dimensional miniplates were created by connecting two miniplates using vertical crossbars to reduce bending. The quadrangular plate’s geometric shape, rather than its length or thickness, increased its stability (thickness 1 mm, length 20 mm, and width 12 mm). Four holes 3D titanium grade 4 miniplates with titanium self-tapping screws in sizes 2 × 8 mm and 2 × 10 mm were used to fix the plates (Fig. 2D).

### Preoperative assessment

Personal, past medical and dental history, and chief complaints were taken. General examination, both extraoral and intraoral examinations, and radiographic examinations were done. A computed tomography (CT) scan was performed.

### Fabrication of 3D-printed models

- Every patient had a CT scan, and the data was imported into Mimics software (MIMICS). Axial section images were used to define image thresholds, removing all soft tissues and highlighting bone and dental tissues. Mandibular bone and teeth were also chosen, and a three-dimensional reconstruction of the segmented mandible, as well as dental hard tissue, was created (Fig. 2A, B).
- When mirroring the alignment of the condyles in the glenoid fossa was considered, then the mandibular bone of the healthy side was mirrored to the fracture side by software to rebuild a perfect reduction model (Fig. 2C). The 3D model of the fractured mandible was printed using a 3D printer. Bone fixation 3D plates were pre-modeled and adapted to the three-dimensional printed model, submitted to sterilization, and were utilized during surgery for bone reduction and fixation (Fig. 2D) [17, 18]. While adapting the 3D plate, we ensured that the horizontal crossbars were positioned perpendicular to the position of the fracture line, while the vertical struts were parallel to it. The plate was contoured to fit the fracture site, with the top crossbar positioned subapically [19]. The miniplate was contoured with the help of pliers to adapt accurately to the surface of the mandible. Because putting one plate in position causes another to follow, this decreased the manipulation time, and as fewer implant materials were used, just one plate and four screws, the overall cost was decreased.

### Surgical phase

The fracture line was exposed through the standard mandibular vestibular approach. Vasoconstrictor injection was done in the submucosal plane to lessen bleeding during incision and dissection. The lower lip was everted, and an anterior incision was made from canine to canine. A scalpel or electrocautery was used to incise the mucosa. Curvilinear and anteriorly extending into the lip, the incision left 10 to 15 mm of mucosa still adhered to the gingiva [20].

The underlying mentalis muscle fibers were incised sharply in an oblique direction to the mandible. It was crucial to keep the mental nerve out of the way. Maxillo-mandibular fixation was temporarily secured, followed by bone reduction into proper anatomical position. The prebent 3D miniplates were fitting the contour of the anatomically repositioned bone fragments following reduction. No plate adjustment was needed, and, in some cases, only a minimal adaptation was done with no significant change in adaptability.

**Fig. 3:**
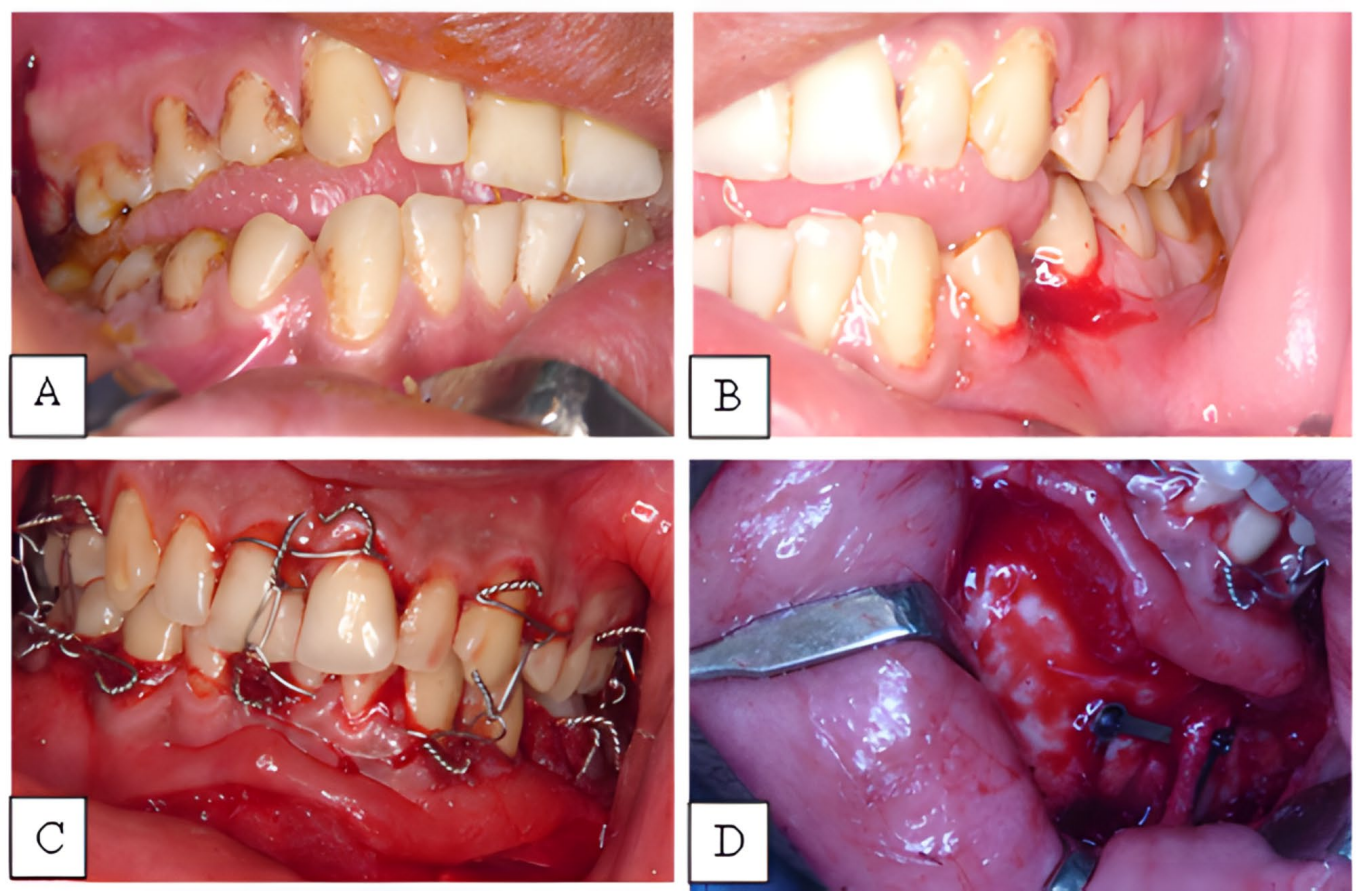
**A-B.** Preoperative occlusions. **C.** intermaxillary fixation. **D.** 3D plate in place after fracture reduction

Except for the anterior region, one layer of closure is sufficient. Three or more deep resorbable sutures must be inserted into the mentalis muscle. Then, a non-resorbable suture is inserted through the origin and insertion of each mentalis, followed by another suture in the midline. Following that, a suture was used to seal the mucosa (Fig. 3).

### Postoperative phase

#### Early postoperative care and postoperative medication

- Antibiotic (amoxicillin clavulanic acid) + analgesic (diclofenac K).
- α-chemo-trypsin ampoules as anti-oedematous once daily for 5 days.
- Cold fomentation for 24 h.
- Povidone Iodine mouth wash 4 times daily.
- Continue oral hygiene measures.

### Follow-up phase

#### Clinical evaluation

##### Postoperative occlusion

The maximal intercuspal position (centric occlusion) was examined to ensure proper occlusal relationship, including molar relation, canine relation, and midline centralization. Any disruption of the occlusal plane, such as an open bite or incorrect tooth contact, was observed. The maximum mouth opening was also assessed.

##### Sensory nerve function [21]

A subjective assessment of the sensory function of the inferior alveolar nerve was done by asking the patient about any alteration in sensation. Objective assessment was done by using a dental probe pressure to determine any sensory changes along the distribution of the mental nerve in comparison to the contralateral side (nociceptive method).

##### Wound healing [21]

The sutured wounds were observed for any indications of wound healing disruption, such as wound dehiscence and plate exposure, and were checked for infection signs and symptoms, such as swelling, redness, heat, pus discharge, and discomfort.

##### Radiographic evaluation [21]

An immediate postoperative CT scan was performed after surgery using the same parameters as the preoperative scan. The DICOM-formatted CT images were imported into the MIMICS software. The mandible was segmented in similarly to the preoperative planning procedure.

**Fig. 4:**
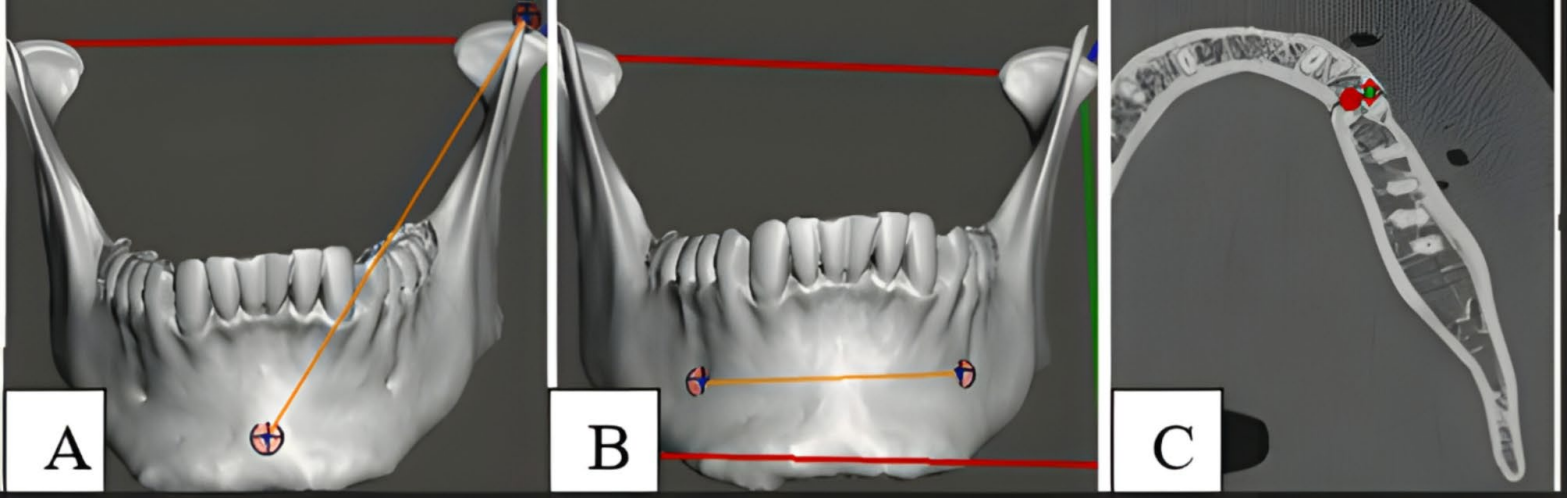
Linear measurements evaluation and bone density. **(A)** Distance from the tip of the coronoid and pogonion. **(B)** distance between the mental foramina. **(C)** calculated Hounsfield Unit (HU) using the RadiAnt DICOM Viewer

To evaluate the accuracy of the virtual plan, the distance was measured between the tip of the coronoid and the most anterior part of the chin (pogonion) (Fig. 4A), and the distance between the mental foramina was also measured (Fig. 4B). The preoperative and postoperative measurements were obtained and statistically compared. Two evaluators independently assessed the results in a blinded manner; two experienced observers conducted all measurements separately, and we evaluated interobserver agreement. We repeated measurements in cases of discrepancies and reached a consensus.

A prospective study following up the patients with another CT scan was taken after 3 months to evaluate bone healing and to estimate the mean bone density at the fracture line. Bone mineral density was calculated in Hounsfield Units (HU) using the RadiAnt DICOM Viewer. Three measurements were taken along the fracture line, and then the mean was calculated per patient (Fig. 4C).

### Statistical analysis

Statistical analysis was carried out using SPSS Statistics software version 29 (SPSS, Inc., Chicago, IL). Quantitative data were tested for normality using Kolmogorov-Smirnov and Shapiro tests. They were not normally distributed; it was described in terms of median, interquartile range, minimum, and maximum. A non-parametric statistical test of significance was applied; the Mann Whitney test was used to compare the parameters between the virtual planning group and the postoperative group. The Wilcoxon signed rank test was used to compare the bone density immediately postoperatively and 3 months after. In all applied statistical tests of significance, *P value* (< *0.05)* was considered significant.

**S**uperimposition of the postoperative 3D model with the preoperative virtual plan was done to confirm adaptability by MeshLab software.

## Results

### Postoperative occlusion

**Fig. 5:**
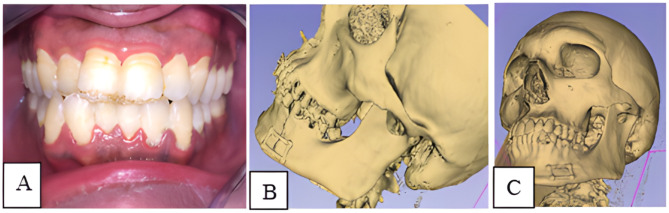
**(A)** Postoperative occlusion. **(B)** Immediate postoperative CT reconstruction. **(C)** Postoperative CT reconstruction after 3 months

All patients were able to achieve normal occlusion and intercuspal relations, and none of them had any occlusal deformities. There was inadequate mouth opening on the immediate postoperative day (< 30 mm), then the normal mean mouth opening was achieved by the time. There was no requirement for any occlusal alteration, selective extraction, or grinding during the follow-up period (Fig. 5A).

### Sensory nerve function

Following surgery, one patient had a postoperative mental nerve disturbance, which began to improve progressively and recovered six weeks after surgery.

### Postoperative wound healing

All the patients reported experiencing pain following surgery; however, it went away a week later. Not a single patient displayed symptoms of infection, dehiscence, or delayed wound healing. There was no movement between the fracture segments and no history of tooth or nerve injury.

### Radiographic evaluation

**Fig. 6:**
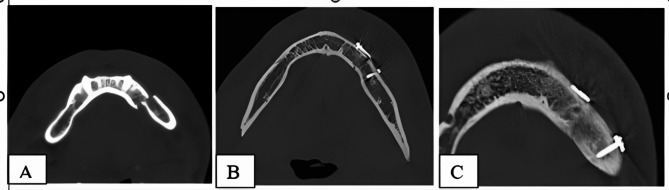
**A**. Preoperative CT axial cuts. **(B)** Immediate postoperative CT axial cuts. **(C)** 3 months postoperative axial cuts showing fixation and healing of the fracture line

Fracture reduction was well achieved, as confirmed by postoperative CT scans and 3D reconstruction. The immediate postoperative and after 3 months CT scan showed good bone segment reduction in all cases and proper alignment of the osseous borders of the mandible (Fig. 5-B, C). Preoperative, immediate postoperative, and 3 months postoperative axial cuts showing fixation and healing of the fracture line (Fig. 6A-C).

### Statistical results

#### Linear measurement evaluation

**Fig. 7:**
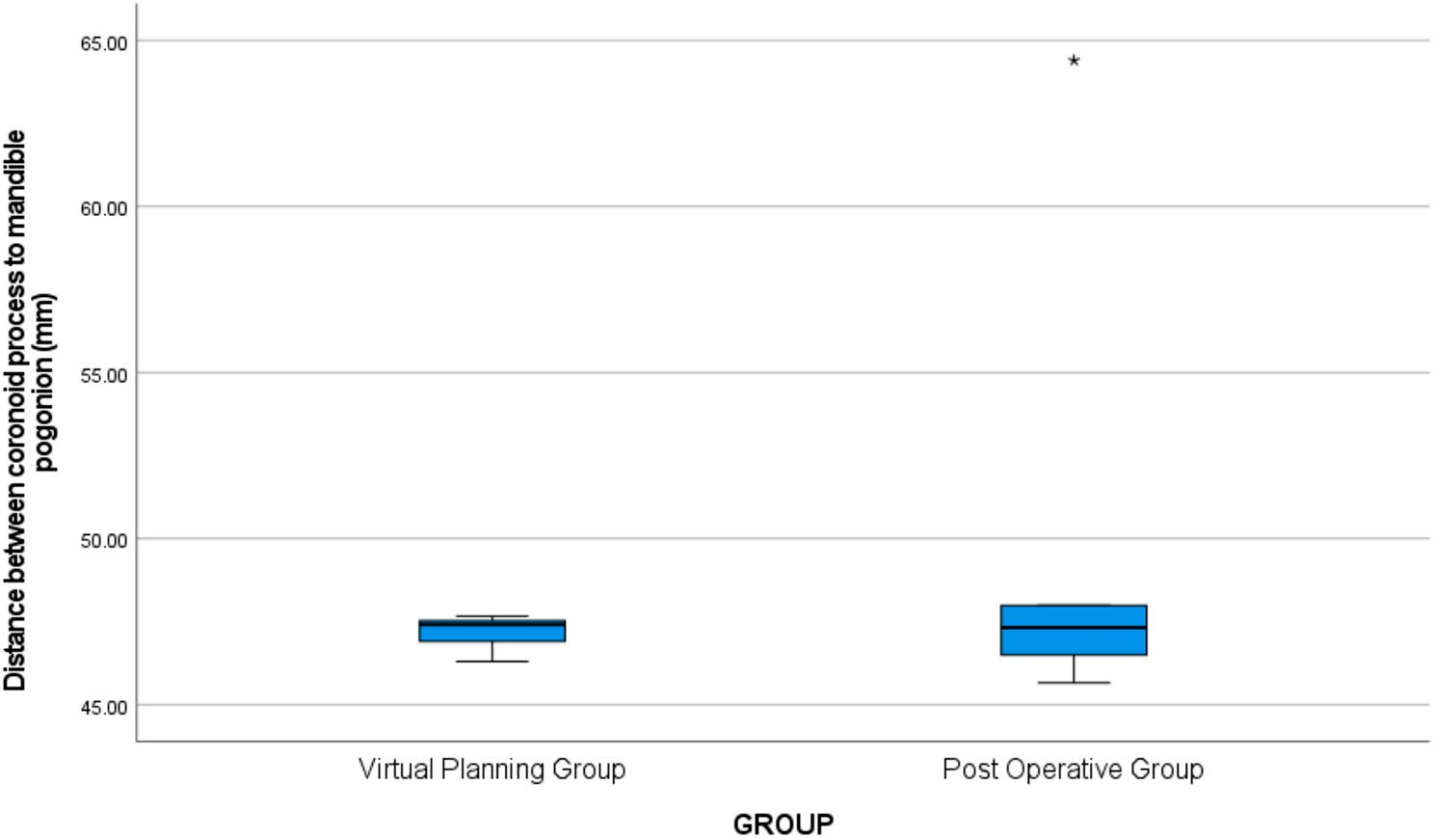
Box plot illustrating the difference in the distance between the coronoid process and the mandibular pogonion between the virtual planning group and the postoperative group

**Fig. 8:**
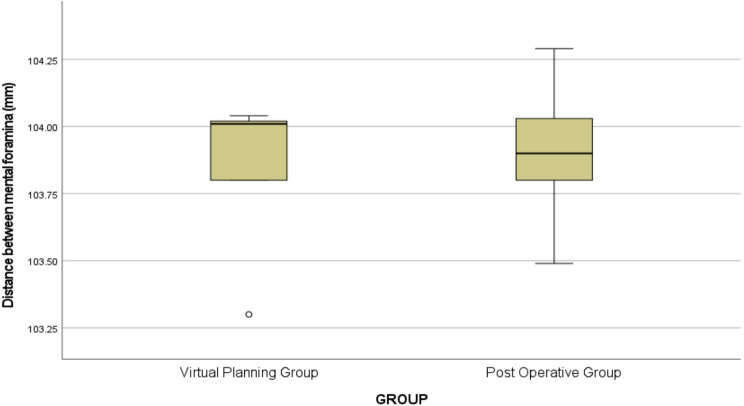
Box plot illustrating the difference in the distance between mental foramina between the virtual planning group and the postoperative group

There was no statistically significant difference in the distance between the coronoid process and the mandible pogonion (mm) between the virtual planning group and the postoperative group [(M1 = 47.41, ranging from 46.30 to 47.67, vs., M2 = 47.32, ranging from 45.66 to 64.40), U = 12.00, *p* > 0.05] as shown in (Table 1) and (Fig. 7). Likewise, there was no statistically significant difference in the distance between mental foramina (mm) between the virtual planning group and the postoperative group [(M1 = 104.01, ranging from 103.49 to 104.04, vs., M2 = 103.90, ranging from 103.49 to 104.02), U = 11.50, *p* > 0.05] as shown in Table 1 and (Fig.  8).

**Fig. 9:**
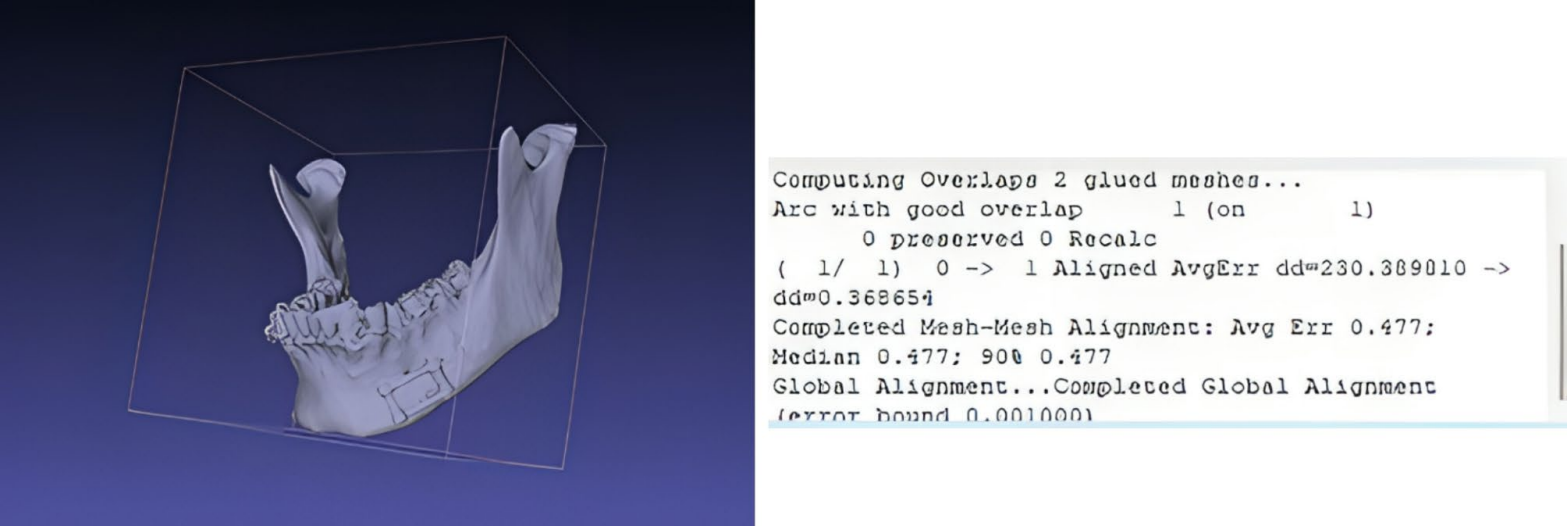
Superimposition of the postoperative 3D model with the preoperative virtual plan. The statistical data obtained are shown in the white panel (dd less than 0.5)

Our results were also confirmed by performing superimposition of the postoperative 3D model with the preoperative virtual plan and revealed good adaptation with distance deviation (dd) less than 0.5 as shown in (Fig. 9).

**Table 1:** Comparison between virtual planning group and postoperative group

	Groups		Statistical test	*P* value
	Virtual Planning Group	Post Operative Group		
Distance between coronoid process to mandible pogonion (mm) Median (Q1- Q3) Min. – Max.	47.41 (46.91–47.53) 46.30–47.67	47.32 (46.50–47.99) 45.66–64.40	12.00	0.917
Distance between mental foramina (mm) Median (Q1- Q3) Min. – Max.	104.01 (103.80–104.02) 103.49–104.04	103.90 (103.80–104.02) 103.49–104.29	11.50	0.834

P value (< 0.05) was considered significant using Mann-Whitney Test

#### Bone density evaluation

**Fig. 10:**
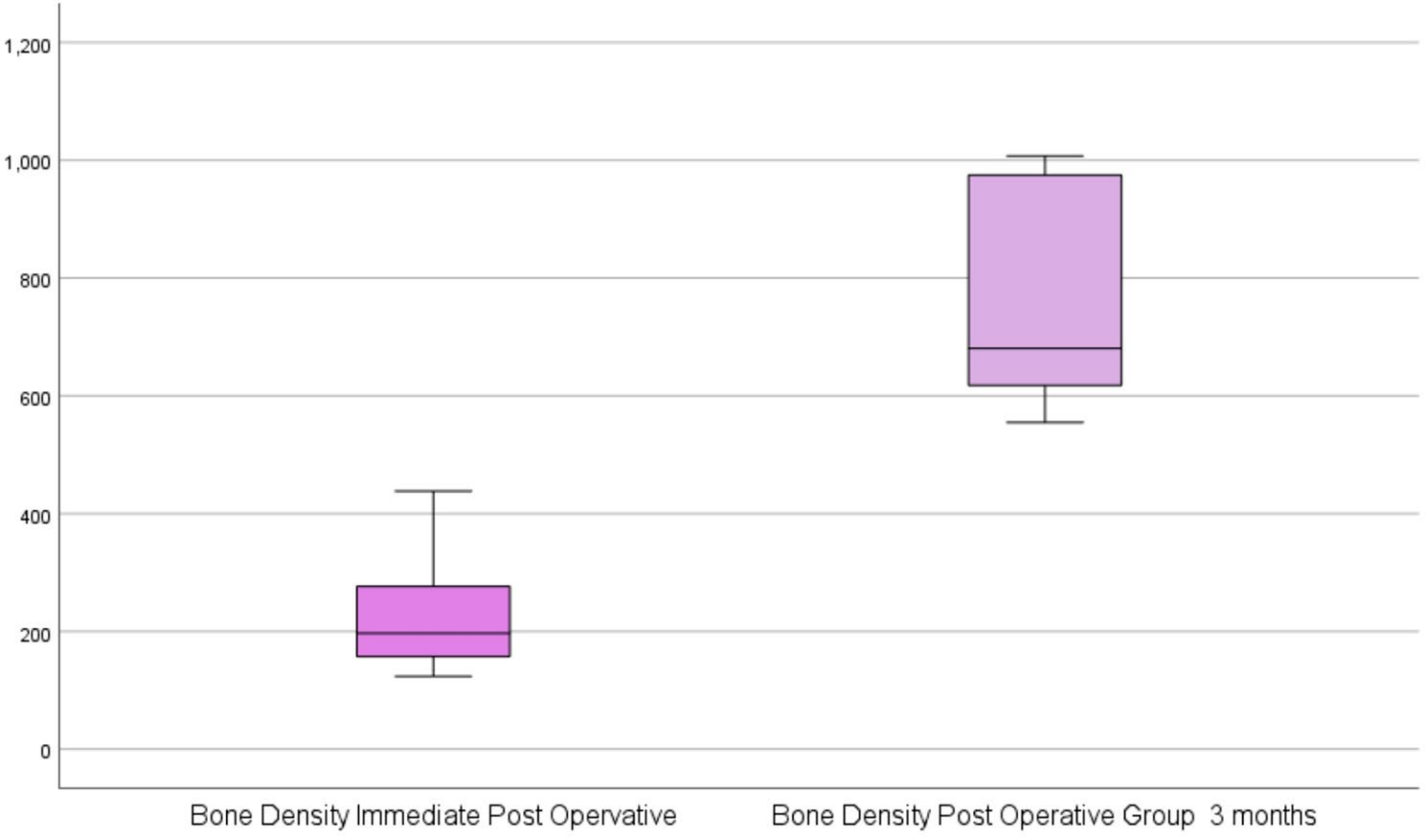
Box plot illustrating the difference in the bone density immediately postoperative and 3 months after

There was a statistically significant difference in the bone density immediately postoperative and 3 months after the operation [(M1 = 197.14, ranging from 124 to 438.4, vs., M2 = 680.55, ranging from 555.01 to 1007.39), Z=-2.201, *p* = 0.028] as shown in Table 2 and (Fig. 10).

**Table 2:** Comparing the bone density immediate postoperative and 3 months after

	Groups		Statistical test	*P* value
	Immediate Post Opervative	Post Operative Group 3 months		
Bone Density Median (Q1- Q3) Min. – Max.	197.14 (157.5–276.5) 124.00–438.40	680.55 (617.8–974.9) 555.01–1007.39	− 2.201	0.028*

P value (< 0.05) was considered significant using Wilicoxon signed Rank Test

## Discussion

Mandibular fractures are among the most frequently encountered fractures by craniomaxillofacial surgeons. Although several techniques for treating these types of fractures have been reported, including various designs of prosthetic devices, accurate anatomical bone reduction cannot be guaranteed. As a result, open reduction and internal fixation is the preferred method for achieving perfect anatomical reduction, particularly in fractures with severely displaced bone segments [22].

The aim of this study was to assess the accuracy of virtual planning and prebinding of 3D miniplates on printed models for fixation of anterior mandibular fractures and to evaluate the clinical outcomes and bone density after fixation.

The results revealed no significant difference between the postoperative three-dimensional image of the mandible and the virtually reduced mandible in the preoperative plan and good bone healing.

The present study revealed the use of virtual planning and 3D printing in mandibular fracture reduction. Other studies that applied the 3D technology and virtual planning for medical interventions ranging from simple surgical procedures to fracture reduction and defect repair had resulted in a significant improvement in clinical outcomes [23, 24].

The mandibular 3D model was printed and used for the adaptation of the 3D miniplate in the present study. This was done by Zhuang H et al., in another study, who printed 3D models using 3D technology, and reconstructive plates were precisely pre-bent before surgery using the 3D-printed mandibular model [25]. Also, stereolithographic models can serve as a template for intraoperative plausibility checks and manual pre-bending of plates prior to surgery as recommended by Probst FA [26].

In this study, mirroring of the contralateral healthy side of the mandible was done. Comparable studies using mirroring of the unaffected side, followed by printing of models, pre-bending of commercial plates or meshes, or patient-specific implant design, reported enhanced accuracy and proven time efficiency [23, 27–30]. Ballard et al. reported that the application of 3D technologies in OMFS can save up to an average of 83 min per surgery when pre-designed surgical guides are used and more than 60 dollars per minute of surgery [31].

In contrast to Saloniemi M et al., who showed that the osteosynthesis of the mandible with patient-specific implants is more difficult since the mandible’s mobility increases the chance of the osteosynthesis loosening and a mirror technique of the face sides is frequently incorrect owing to its intrinsic lack of symmetry [32].

The benefit of using virtual surgical planning before open reduction and internal fixation was reported by Maloney & Rutner. The shorter surgical time and duration of general anesthesia, as well as the more precise adaptation of the hardware to the bony topography of the mandible when compared to plate adaptation performed intraoperatively, were reported [33]. The placement of 3D miniplates was simple and easy for use in our study.

A patient-specific custom plate was not applied in this research because the time required for production was not worth it when a prebent plate could be prepared in less time and provided comparable benefits. Case studies demonstrated the advantages of using virtual surgical planning preoperatively and having the hardware readily available at the time of fracture exposure [33].

Concerning accuracy, there was no significant difference between the preoperative and postoperative measurements and between preoperative reduced mandible and postoperative three-dimensional mandible images. These results may be due to the plate’s quadrangular geometry, which ensures 3D stability of the fracture sites by providing good resistance to torque forces, avoiding the need for maxillomandibular fixation, and ensuring early restoration of parasymphyseal function [34].

The absence of significant differences between preoperative and postoperative measurements supports the accuracy of the digital workflow but does not inherently indicate superiority over conventional methods.

This was in contrast to the results of Mishra N et al., concluded that the clinical results of 3-D miniplates and traditional miniplates do not differ much. With similar rates of problems, either fixation technique can be effectively utilized to treat mandibular fractures [19].

Advantages of 3D miniplates in comparison with conventional plates were mentioned by Malhotra K et al., that only one plate and four screws are fixed and the 3D miniplate system requires less implant material in the symphysis and parasymphysis region than miniplates, which have two plates, and eight screws attached. Also, when comparing 3-D miniplates to those from the same manufacturer, the total treatment cost is cut in half [35].

Bhatt V et al., found that even though the 3D plate is somewhat more expensive than traditional 2D miniplates, however, because only one plate is needed for anterior mandibular fractures, fewer numbers of screws are used. This information renders the increased cost useless. The use of 3D plates further lowers operational time costs [36].

These findings were consistent with a study done by Singh et al., who compared 3D miniplates to traditional ones and discovered that the 3D miniplate system operated for longer in the angle region and less in the symphysis/parasymphysis region [37].

Accuracy was assessed in our study by measuring distances between anatomical landmarks of the mandible in an anteroposterior and transverse direction. Annino DJ et al., recommended assessing accuracy using the mandibular condyle as an anchor and a 3D model overlay in his study about virtual planning and 3D-printed guides for mandibular reconstruction [38].

Our results were also confirmed by performing superimposition of the postoperative 3D model with the preoperative virtual plan one and revealed good adaptation. This method was used by.

Committeri U et al., who did a superimposition of the postoperative 3D model obtained from a CT scan after surgery and the 3D virtual model for evaluation of virtual planning and mirroring methods in relation to open reduction and internal fixation for fractures of the zygomaticomaxillary complex [39].

The stability of the postoperative reduction in the present study may be due to the fixation by the 3D miniplate. Budhraja NJ et al. clarified that when the mandible is in function, primary forces of concern are bending, vertical displacement, and shearing. The vertical bars connecting the two horizontal bars in a 3D plate resist bending forces. Instead of distributing stresses along a single line, the plate’s box structure disperses them across a surface area, providing greater three-dimensional stability resisting shearing, bending, vertical displacement, and torsion forces. As a result, three-dimensional stability is gained, hence the name 3D plate [40]. The statistically significant increase in bone density may be partially attributed to the natural bone healing process, in addition to the stability provided by the fixation method.

In the present study, enhancement in bone density revealed by increased Hounsfield Units was significant, and this may be due to the previously mentioned design of fixation of 3D plates. In the present study, the postoperative following period was continuous till 3 months, and this is recommended by T. Kawai et al., who recommend follow-up radiographic examination to confirm clinical judgment during the fifth week after a mandibular fracture in patients less than 18 years of age and the ninth week for older patients [41].

When the occlusion was examined postoperatively, we discovered that there was no occlusal disturbance and no mobility of the fractured segments in all cases. Our findings were similar to those of Gear et al., and Alkan et al., who demonstrated that 3D strut plates have superior compression load resistance than Champy’s technique [42, 43]. Also, Mohd et al., found that patients treated with 3D plates had a lower incidence of occlusal discrepancy than Champy’s miniplates, though the difference between groups was statistically insignificant [34].

Malhotra K et al., who compared the 3D miniplate systems to traditional miniplates in the treatment of mandibular fractures, concluded that after the fracture is fixed, both systems exhibit sufficient stability [35]. This may help explain why conventional miniplates are still widely used and effective.

Patients with comminuted fractures were excluded in this study, and this was considered a limitation for using 3D miniplates. Another study revealed that a limitation of 3D plates is their placement over comminuted and oblique fractures. As a result, Champy’s miniplates have an advantage over 3D plates in these situations [34].

In the parasymphysis angle region, 3D plates were found to be better than miniplates; however, in cases of oblique fractures and body fractures that are in and around the mental foramen, placement of these plates is not possible without injuring the neurovascular bundle [44]. To overcome this limitation around the mental foramen, an indigenous custom-made detachable 3D titanium plate was designed [45].

Another problem with this study was that it had a small sample size. This could be because of the exclusion criteria, which limited how the 3D miniplate could be used. Another problem is that the follow-up period was too short, which could be because of difficulties in patients recall. Longitudinal studies with larger sample sizes are recommended for future studies. Furthermore, another limitation was the absence of a control group and the need for future studies with control groups to further validate the finding.

Despite their effectiveness, stereolithographic models can only provide indirect guidance and cannot confirm “real-time” fracture reduction. A revolutionary technique for transferring virtual planning to actual surgery is the surgical guide, which is the result of combining CAD/CAM and fast prototyping technology [46].

Although 3D printing technologies are used extensively, they have limitations in use. Despite the potential cost constraint, the cost of 3D technology, in terms of devices, materials, and software, is continuing to fall [47]. Another potential limitation is the time required to create a 3D-printed model. This includes the time required to capture anatomical scans, create a virtual 3D prototype, layer by layer 3D print the material, and finally modify the final structure. This can also vary between 1 and 24 h, depending on the size of the object and the printing resolution [48].

## Conclusion

Based on the clinical and radiographic outcome of our study, it is concluded that digital workflow, including virtual planning and 3D printing, provides an accurate method for the reduction and fixation of anterior mandibular fractures. Also, 3D miniplates provide a good option for symphyseal and parasymphyseal fractures, as evidenced by increased bone density, despite their limitations, as in some cases like comminuted fractures and fractures in and around the mental foramen. The small sample size and the short period follow-up were also considered limitations in this study. We recommend further longitudinal studies with a larger sample size with a control group to accurately evaluate the virtual planning, 3D printing, and 3D miniplates in the treatment of anterior mandibular fractures.

## Data Availability

Due to [privacy/ethical/confidentiality] reasons, the research data are not publicly available but can be provided upon request. The datasets used and/or analysed during the current study are available from the corresponding author on reasonable request.

## Abbreviations

3D: Three-dimensional
CT: Computed tomography
HU: Hounsfield Unit
IRB: Institutional Review board
IORG: Institutional Official Registration with the Office for human Research Protections (OHRB)
CAS: Computer- assisted surgery
MRI: Magnetic resonance imaging
DICOM: Digital Imaging and Communications in Medicine
STL: Standard Tessellation Language
CAD: Computer- aided design
Mm: Millimeters
dd: Distance deviation
